# Detection of SARS-CoV-2 from raw patient samples by coupled high temperature reverse transcription and amplification

**DOI:** 10.1371/journal.pone.0241740

**Published:** 2020-11-02

**Authors:** Johannes W. P. Kuiper, Timo Baade, Marcel Kremer, Ramon Kranaster, Linda Irmisch, Marcus Schuchmann, Johannes Zander, Andreas Marx, Christof R. Hauck

**Affiliations:** 1 Lehrstuhl Zellbiologie, Universität Konstanz, Konstanz, Germany; 2 Konstanz Research School Chemical Biology, Universität Konstanz, Konstanz, Germany; 3 Labor Dr. Brunner, Konstanz, Germany; 4 myPOLS Biotec GmbH, Konstanz, Germany; 5 Klinikum Konstanz, Konstanz, Germany; 6 Lehrstuhl Organische Chemie/Zelluläre Chemie, Universität Konstanz, Konstanz, Germany; Florida Atlantic University, UNITED STATES

## Abstract

SARS-CoV-2 is spreading globally with unprecedented consequences for modern societies. The early detection of infected individuals is a pre-requisite to contain the virus. Currently, purification of RNA from patient samples followed by RT-PCR is the gold standard to assess the presence of this single-strand RNA virus. However, these procedures are time consuming, require continuous supply of specialized reagents, and are prohibitively expensive in resource-poor settings. Here, we report an improved nucleic-acid-based approach to detect SARS-CoV-2 with the ability to detect as little as five viral genome equivalents. The approach delivers results without the need to purify RNA, reduces handling steps, minimizes costs, and allows evaluation by non-specialized equipment. The use of unprocessed swap samples is enabled by employing a heat-stable RNA- and DNA-dependent DNA polymerase, which performs the double task of stringent reverse transcription of RNA at elevated temperatures as well as PCR amplification of a SARS-CoV-2 specific target gene. As results are obtained within 2 hours and can be read-out by a hand-held LED-screen, this novel protocol will be of particular importance for large-scale virus surveillance in economically constrained settings.

## Introduction

As of September 2020, severe acute respiratory syndrome coronavirus 2 (SARS-CoV-2) is responsible for more than 32.7 million COVID-19 cases and associated with more than 991 000 fatalities (WHO; COVID-19 Weekly Epidemiological Update [Accessed 30. September 2020]. Available from: https://www.who.int/emergencies/diseases/novel-coronavirus-2019/situation-reports). Within a few months of its emergence, this infectious agent has spread globally and is derailing societies worldwide. SARS-CoV-2 is an enveloped plus-strand RNA virus of the beta-coronavirus genus with closely related strains circulating in bats and some other mammals indicating a zoonotic origin [1, 2]. Upon host cell infection, beta-coronaviruses generate a minus-strand RNA copy of their genome by a virus-encoded RNA-dependent RNA-polymerase [3]. Furthermore, the virus directs the synthesis of several subgenomic minus strand RNAs, which are complementary to the 3’ end of the viral genome and which encode several non-structural proteins including the N gene [3]. Therefore, infected cells harbor plus and minus strand RNAs of the coronavirus, with an overabundance of transcripts derived from the 3’ end of its genome [4, 5]. As with other RNA viruses, confirmation of SARS-CoV-2 infection is based on the molecular biological detection of the viral genome and its transcripts in patient samples by nucleic acid amplification techniques (NAATs) [6]. To allow sensitive and accurate detection of viral ribonucleic acids, primary patient samples such as nasopharyngeal swabs, sputum or stool are further processed to isolate the total RNA. Using reverse transcriptase, the RNA is then reverse transcribed into DNA. Next, the DNA is PCR-amplified by a thermo-stable DNA-dependent DNA polymerase using specific primers and probes to detect the presence of SARS-CoV-2 sequences.

Facilitated by genome sequencing and rapid information sharing, such a NAAT-based diagnostic surveillance of SARS-CoV-2 has been in place right from the beginning of the COVID-19 pandemic [7, 8]. However, the current methodology is laborious, requires multiple handling steps and expensive consumables, resulting in a costly diagnostic procedure, which can constrain COVID-19 testing in economically tight settings. Furthermore, the overwhelming worldwide demand for some of the same reagents (such as those needed for RNA isolation) and diagnostic kits has produced shortages and unnecessarily delayed or restricted testing. From a clinical point of view, rapid testing and early decision making on further isolation measures for patients and health care workers remains a critical issue.

We have developed a thermostable DNA polymerase, which can mediate DNA synthesis from both RNA as well as DNA templates [9]. By targeted modifications, we have further improved the accuracy and processivity of this enzyme [10], which lays the foundation of the commercialized Volcano3G formulations. We reasoned that such a bi-functional enzyme might allow us to improve and simplify the detection of RNA viruses. Here we show that Volcano3G can be used in a coupled high-temperature reverse transcription and amplification reaction to detect SARS-CoV-2 with high sensitivity and specificity. Moreover, our findings demonstrate the usefulness of such a thermo-stable RNA- and DNA-reading DNA polymerase to simplify COVID-19 diagnostics. Most importantly, we can show that this robust enzyme allows detection of SARS-CoV-2 directly from unprocessed patient material. Accordingly, this streamlined procedure, which does not depend on limited reagents, nor requires expensive equipment, is poised to enable large scale SARS-CoV-2 surveillance in a multitude of resource- or time-constrained settings.

## Results and discussion

### A RNA- and DNA-reading heat-stable polymerase reverse transcribes and amplifies viral RNA

**Fig 1:**
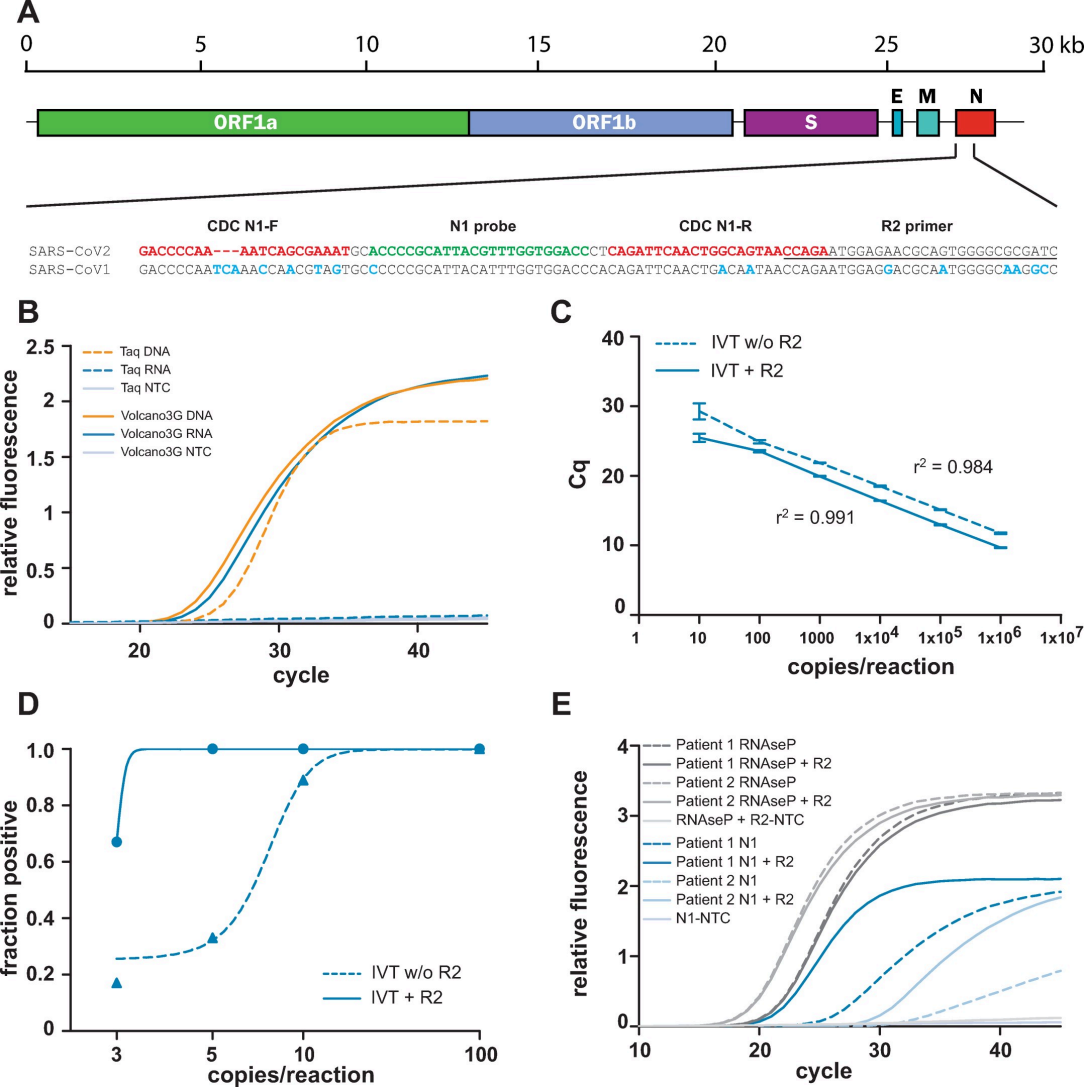
A RNA- and DNA-reading heat-stable polymerase reverse transcribes and amplifies viral RNA. **A)** Schematic overview of the SARS-CoV-2 genome. The target sequences for the N1 primers and probe are marked in red and green, respectively. The R2 primer binding sequence is underlined. Sequences divergence between SARS-CoV-2 and SARS-CoV-1 genomes are highlighted in blue. **B)** Performance of Volcano3G polymerase was compared to Taq polymerase using plasmid DNA or in vitro transcribed RNA as template (5000 viral genome equivalents). **C)** Determination of the linear dynamic range for the Volcano3G protocol with or without an additional primer (R2) for optimized reverse transcription at a final concentration of 250 nM. In vitro transcribed RNA containing the Sars-CoV-2 N amplicon was serially diluted in the range from 1x10^6^ copies to 10 copies. **D)** Limit of detection (LOD) was assessed with serial dilutions ranging from 20 to 1 copy per reaction (n = 6 for each dilution). The fraction of positive reactions (y-axis) were plotted against the log-transformed number of RNA copies per reaction. Addition of R2 primer enhances the performance at lower copy-numbers. **E)** Amplification curves showing the performance of Volcano3G on isolated RNA from two COVID-19 patients in presence or absence of R2.

Evidence of an acute SARS-CoV-2 infection depends on the detection of viral RNA species in patient samples, which necessitates reverse transcription of RNA followed by PCR amplification of the resulting DNA. To confirm that the Volcano polymerase is able to perform both of these steps, we employed an *in vitro* transcribed synthetic SARS-CoV-2 RNA template covering a ~750 nt stretch within the 3’-end of the viral genome (Fig 1A). This target region is also included in the CDC panel of primers (Division of Viral Diseases, National Center for Immunization and Respiratory Diseases, Centers for Disease Control and Prevention, Atlanta, GA, USA; https://www.cdc.gov/coronavirus/2019-ncov/lab/rt-pcr-panel-primer-probes.html; accessed on 5.5.2020). When 5000 genome equivalents of the purified, in vitro transcribed viral RNA was used as a PCR template for a generic, heat-stable DNA-dependent DNA polymerase (Taq DNA polymerase) no amplification occurred, demonstrating the absence of a contaminating DNA template (Fig 1B). In contrast, when Volcano3G was employed, the expected amplification product was obtained, confirming that the Volcano3G polymerase can read the RNA template to produce and amplify a specific DNA sequence (Fig 1B). Clearly, the Taq DNA polymerase was able to yield an amplicon, when DNA instead of RNA was used as a template (Fig 1B). Not surprisingly, the Volcano3G polymerase was able to operate with primers and probes supplied by other manufacturers targeting the same region of the SARS-CoV-2 N gene (S1A and S1B Fig). Due to the thermostability of the Volcano3G polymerase, this RNA-reading enzyme could perform, in contrast to other widely used enzymes, the reverse transcription under stringent, high-temperature conditions. Reverse transcription at elevated temperatures could help to overcome stable RNA-secondary structures present in beta-coronavirus genomes [11, 12]. To take advantage of this particular feature of the Volcano3G polymerase, we added an extended, high-melting temperature virus-specific primer (R2) to the reaction, and adjusted the PCR protocol to include a high-temperature reverse transcription step. As the 3’-end of the viral genomic plus-strand RNA and its minus-strand complementary sequences are the most abundant nucleic acids in coronavirus-infected cells, we focussed on the coronavirus N gene, which is located at the very 3’-end of the SARS-CoV-2 genome [13]. Furthermore, the amino terminus of the N protein varies greatly between different human-pathogenic beta-coronavirus isolates (S1C Fig), making this region an ideal target for SARS-CoV-2-specific primers. Accordingly, the high-temperature RT primer R2 was designed to be complementary to sequences at the 5’ end of the N gene (Fig 1A). The addition of the R2 primer consistently increased the performance of the Volcano3G reaction resulting in lower cq-values over a wide range of template concentrations (Fig 1C). The high-temperature reverse transcription afforded by Volcano3G polymerase and the R2 primer worked at temperatures >70°C (S1D Fig). While the optimal elongation temperature for the purified RNA template was at the lower end of these temperatures (S1D Fig), a slightly elevated temperature (75°C) was chosen with more complex RNA samples to achieve a balance between stringency and polymerase acitivity. Under these experimental conditions, addition of R2 lowered the limit of detection (LOD) to five copies of the viral genome equivalent (Fig 1D). To assess, if this adapted procedure allows the sensitive detection of SARS-CoV-2 in patient material, we used RNA isolated from nasopharyngeal swabs of two confirmed COVID-19 cases. In both samples, amplification of the human RNAseP transcript demonstrated the integrity of the samples and resulted in similar cq-values in the presence or absence of the additional R2 primer, while the non-template control (NTC) gave no signal (Fig 1E). Most importantly, the Volcano3G polymerase detected viral RNA in both samples, but produced exceptionally low cq-values upon addition of the R2 primer, suggesting that the high-temperature reverse transcription by the thermo-stable RNA-reading DNA polymerase opens an additional window of opportunity for hypersensitive detection of viral RNA. Together, these initial results encouraged the further exploration of the Volcano3G polymerase for facilitating the detection of SARS-CoV-2 RNA.

### SARS-CoV-2 detection by high-temperature RT-PCR in a patient cohort delivers results consistent with the standard procedure

**Fig 2:**
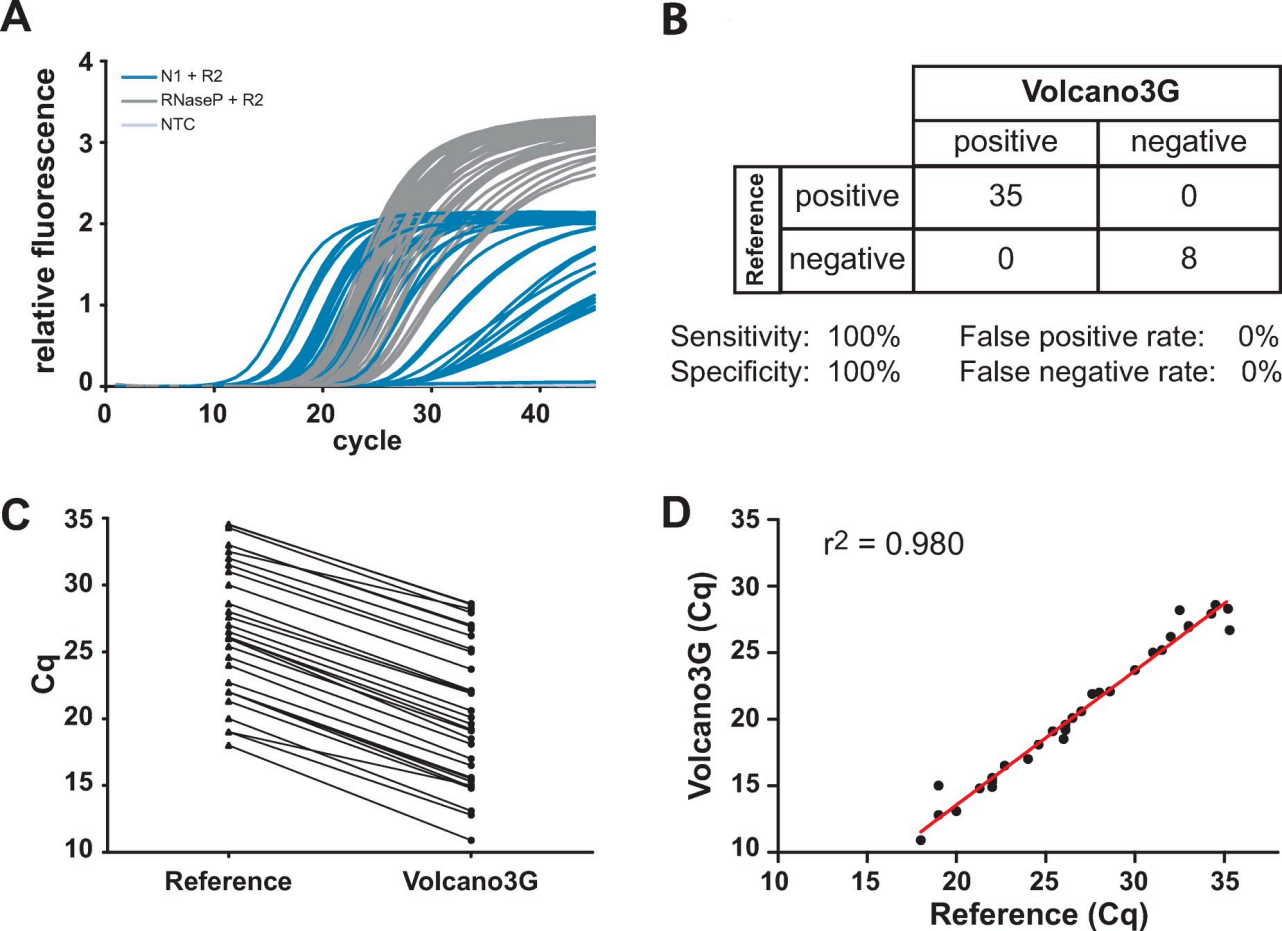
SARS-CoV-2 detection by high-temperature RT-PCR in a patient cohort delivers results consistent with the standard procedure. **A**) RNA was isolated from nasopharyngeal- and throat swab samples (n = 43) and SARS-CoV-2 and RNAseP were detected using the Volcano3G protocol. N1 amplicon (blue), RNaseP gene (gray). Water was used as a non-template control (light gray). **B)** Identical samples were processed in parallel in an accredited diagnostic lab using the Allplex 2019-nCoV assay from Seegene. Direct comparison of assay results reveals 100% concordance of Volcano3G with the reference assay. **C)** Cq values obtained with Volcano3G were lower than those obtained with the reference assay (ΔCq = 6.4 +/- 0.78). **D)** For each positive patient sample, the Cq values obtained with both assays were plotted against each other for linear regression analysis. A highly significant correlation of Volcano3G with the reference assay was observed (r^2^ = 0.98, p<0.0001).

To evaluate the potential of the high-temperature RT-PCR protocol using Volvano3G for the detection of viral RNAs in patient material, we assessed the presence of SARS-CoV-2 in RNA isolated from a small cohort of COVID-19 suspected cases. RNA was isolated from nasopharyngeal swabs and the isolated nucleic acid was then evaluated in parallel by i) a commercial in vitro diagnostic kit (Allplex, Seegene) and ii) high-temperature RT-PCR with Volcano3G. Of the 43 samples, the Allplex-assay detected the SARS-CoV-2 N gene in 35 samples, while 8 samples remained negative (Fig 2A). The eight negative samples also did not yield amplicons in the Allplex-assay for the RdRp or the E gene (data not shown). When the 43 RNA samples were employed in high-temperature RT-PCR with Volcano3G polymerase, the identical results were obtained, showing complete consistency with regard to positive and negative outcomes (Fig 2A and 2B). Pairwise comparison of the cq-values obtained for the isolated RNA samples in detecting the N gene revealed that the high-temperature RT-PCR with Volcano3G resulted in lower cq-values throughout all samples suggesting a slightly increased sensitivity (Fig 2C). In addition to the resolution of RNA secondary structures, the increased sensitivity might be due to the fact that the high temperature reverse transcription step involves several cycles, which allow initial highly stringent amplification of viral target genes. Though the high temperature RT-PCR with Volcano3G was slightly more sensitive, the results correlated extremely well over a wide range of cq-values with the results obtained by the commercial assay (r^2^ = 0.980, p<0.0001, Fig 2D). Together, these findings suggest that high temperature RT-PCR with Volcano3G could be an additional option to rapidly detect RNA-virus genomes with high specificity and sensitivity.

### High-temperature RT-PCR using Volcano3G polymerase allows SARS-CoV-2 detection from unprocessed patient samples

**Fig 3:**
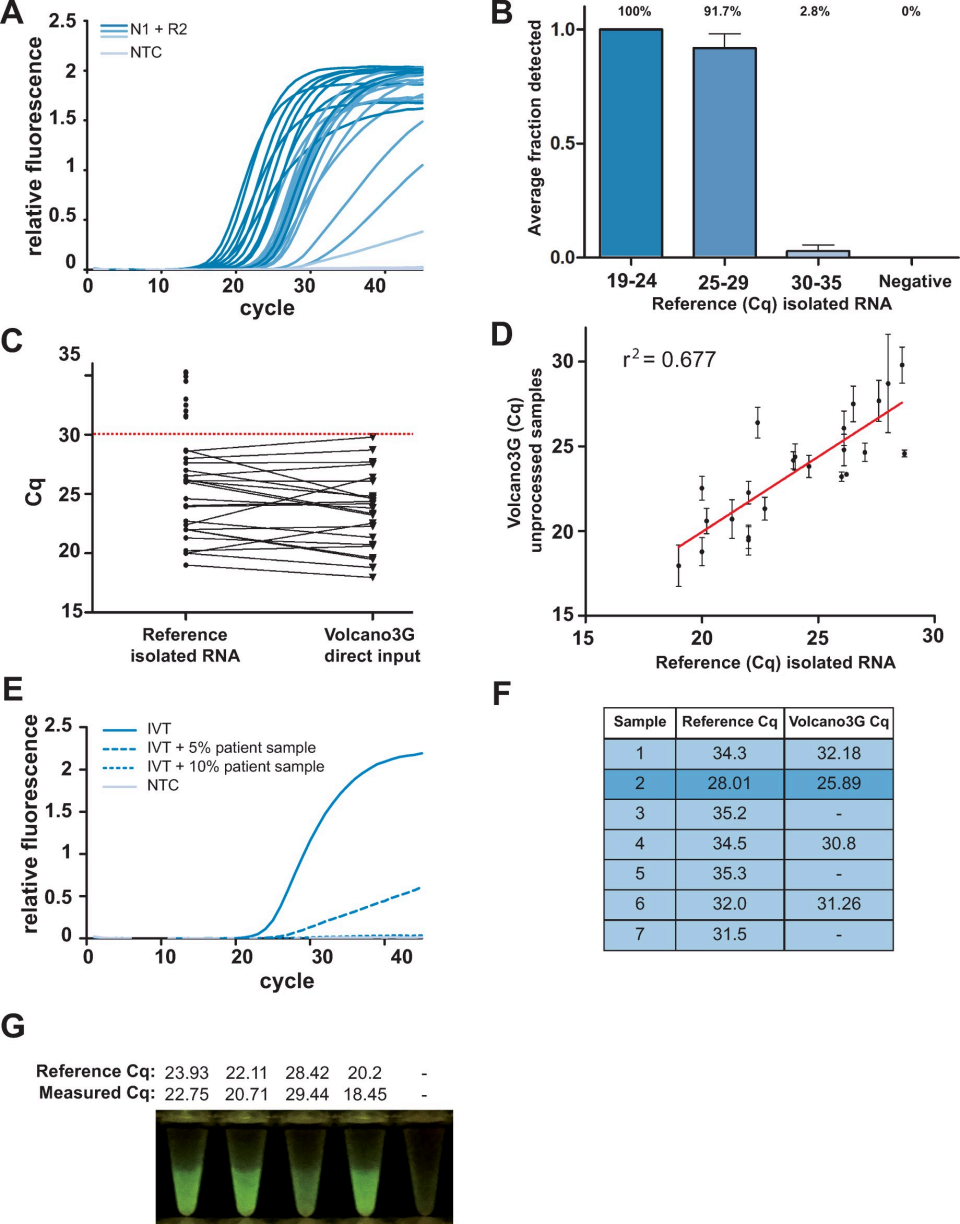
High-temperature RT-PCR using Volcano3G polymerase allows SARS-CoV-2 detection from unprocessed patient samples. **A)** Nasopharyngeal- and throat swab samples (prepared in water) were added directly as template for RT-qPCR using the Volcano3G protocol. Representative amplification curves of patients with high (dark blue), medium (medium blue) and low Cq as well as negative patients (light blue) are shown. **B)** RNA was isolated from the remaining patient material and analysed in an accredited diagnostic lab using the Allplex 2019-nCoV assay from Seegene. The samples were divided in 3 groups based on cq (<24, 24–30 and >30) and each sample was analysed repeatedly (3–4 times) by high-temperature RT-PCR. The bar diagram depicts the average frequency of detection (± SEM) for each group (patient samples in each group: cq < 24 n = 12; cq 24–29 n = 12; cq > 30 n = 9 and negative samples n = 12). **C)** The cq values of each patient sample are compared between the reference protocol and the Volcano3G direct approach. Dotted red line indicates the cut-off, were the direct assay looses sensitivity. **D)** For each positive patient sample, the Cq values obtained in 3–4 repetitions with the high-temperature RT-PCR (Volcano3G direct input; mean +/- sd) were plotted against the cq-values obtained with the standard RT-PCR on isolated RNA for linear regression analysis (r^2^ = 0.779, p<0.0001). **E)** RT-PCR analysis of 100 copies of in vitro transcribed RNA spiked with varying amounts of pooled patient material from 5 confirmed negative patients. **F)** 10 swabs were eluted a second time according to our optimized protocol (low elution volume, protease K treatment and additional MgCl_2_). In addition, 10 confirmed negative swab samples were included. **G)** 5 Volcano3G reactions from the cohort of unprocessed samples presented in Fig 3A (in low-translucent white tubes) were photographed on a blue light transilluminator. Reference Cq values (Seegene Allplex on purified RNA) and Cq values obtained with Volcano3G using unprocessed samples (measured Cq) are depicted above each tube.

Besides enhanced stringency and possible resolution of RNA secondary structures, we speculated that high-temperature RT-PCR might promote viral lysis and allow the detection of SARS-CoV-2 RNA directly from unprocessed patient samples. To this end, we employed a second cohort of samples, where each nasopharyngeal swab was initially resolved in 300 μl of distilled water. While 150 μl of these diluted samples were used for RNA extraction and standard RT-PCR, 12 μl of the diluted sample were directly employed in high-temperature RT-PCR with the Volcano3G polymerase. Importantly, even with this raw patient material, the high-temperature RT-PCR yielded clear-cut results without increasing the background (Fig 3A). Each sample was analysed repetitively in three to four separate high-temperature RT-PCR runs with the Volcano3G polymerase and the results obtained were compared to the standard RT-PCR from purified RNA of the identical samples. Importantly, all negative samples were consistent between the two approaches suggesting that there is no increased risk of producing false positive results, when using the unprocessed patient samples (Fig 3B). Moreover, 100% of the samples showing cq-values of ≤ 24 for the SARS-CoV-2 N gene in the standard RT-PCR assay with purified RNA were correctly identified by the Volcano3G high-temperature RT-PCR employing raw patient samples (Fig 3B and 3D). Also, 10 out of 12 samples showing cq-values >24 and ≤ 30 for the SARS-CoV-2 N gene in standard RT-PCR from purified RNA were detected by direct high-temperature RT-PCR from unprocessed samples in all replicates (Fig 3B). The remaining 2 samples of this set were positive in 1 or 3 out of four replicates, respectively (Fig 3B), Samples yielding cq-values above 30 in the standard RT-PCR following RNA extraction, which would represent a low viral load, were only rarely detected as positive, when patient samples were used without further processing (Fig 3B). None of those patients, from which samples with low viral loads (cq-value >30) were obtained, was hospitalized and all of them showed only mild symptoms including sore throat and rhinitis. It is important to stress that clinical studies of viral dynamics in patients have shown that SARS-CoV-2 titers in swab samples are most often high during the initial phase of the infection, even before onset of symptoms [14–17]. In contrast, low titers are typically seen at the later phase of the infection and can even be detected after the resolution of symptoms [18]. Observations from laboratory-confirmed SARS-CoV-2 transmission pairs indicate that transmissability is highest around the onset of symptoms, when viral titers in nasopharyngeal swabs are high [19], and that infectiousness of clinical samples towards cultured cells is negligible, if cq-values are above 24 [20]. Indeed, virological assessment of patient-derived swap material has shown, that samples yielding cq-values > 30 in regular RT-PCR from purified RNA (corresponding to ~10^6^ viral copies/ml of sample) do not allow productive infection of cell cultures [21]. Based on these findings, the simple and rapid detection of people sustaining high SARS-CoV-2 titers as afforded by high-temperature RT-PCR of unprocessed patient material might be sufficient to break chains of viral transmission.

Interestingly, for most positive samples detected by the high-temperature RT-PCR with Volcano3G, the cq-values were lower compared to the standard RT-PCR (Fig 3C and 3D), indicating that the detection of SARS-CoV-2 from unprocessed patient material is not limited by the sensitivity of this direct approach. As unprocessed swab material might contain numerous patient-derived proteins, mucus, or membrane fragments, we speculated that substance(s) within these raw samples could interfere and inhibit the detection of SARS-CoV-2 when only a low viral load is present. Therefore, we tested the inhibitory potential of the eluted swab material. Using the in vitro transcribed viral RNA as a template, we spiked the Volcano3G reaction mix with increasing amounts of swab-derived material (Fig 3E). Importantly, there was a clear dose-dependent inhibition of the amplification reaction due to the material eluted from the swabs (Fig 3E and S2 Fig). The extent of inhibition showed variability between individual samples (S2 Fig), with some samples leading to complete inhibition of viral detection at 10% added swab material (Fig 3E). Especially, for samples containing low viral titers this could significantly elevate the LOD. Therefore, we supplemented the distilled water used for eluting the nasopharyngeal swabs with betaine, BSA, carrier RNA, DTT or treated them with proteinase K or combinations of these reagents, with the idea that these substances might be able to alleviate the inhibition due to inhibitory proteins or RNA degrading or oxidizing agents. Indeed, several of the treatments enhanced the detection of SARS-CoV-2 from an unprocessed patient sample demonstrating that it is possible to partially overcome the inhibitory effect of the raw material (S3A and S3B Fig).

As patient samples are expected to contain molecules that sequester magnesium ions (e.g. DNA, proteins) and calcium ions and therefore compete with magnesium for binding to polymerases, we hypothesized that increasing the concentration of MgCl_2_ might augment the PCR reaction. Indeed, increasing concentrations of MgCl_2_ lowered the threshold of detection of RNA template in presence and absence of spiked-in (SARS-Cov-2 negative) patient material (3.2 and 2.8 cq-values, respectively) (S3C Fig). Since treatment of nasopharyngeal samples with proteinase K lowered the detection threshold (S3B Fig), we next assessed its combinatory effect with higher concentrations of MgCl_2_. Especially in reactions with low template numbers, a positive effect of proteinase K treatment together with addition of MgCl_2_ was observed (S3D Fig). Proteinase K treatment is normally performed at relatively high temperatures (50°C-70°C) to promote protein unfolding and enhance enzyme activity. Afterwards, inactivation of proteinase K is required to protect the polymerase from proteolysis. Though inactivation of proteinase K is commonly achieved by heat treatment (5–10 min at 95°C) these high temperatures also promote RNA degradation, especially in the presence of divalent cations (S3E Fig). To circumvent the thermal inactivation of proteinase K we included instead the serine protease inhibitor phenylmethylsulfonyl fluoride (PMSF) in our final optimized protocol (S3F Fig). To re-evaluate 10 nasopharyngeal swabs, which had shown low viral titers on initial testing (conventional RT-PCR cq > 30), we eluted these swabs again in 50 μl RNAse-free water supplemented with 10 ng/μl carrier RNA and stored for 1 month at -20°C. This small volume (compared to 350 μl for the cohort in Fig 3A and 3B) was chosen to increase the concentration of eluted virus from these low-titer samples. These re-eluted low-titer samples were subjected to our optimized protocol (S3F Fig). Again, we did not observe any false-positives, but were able to detect 4 out of 10 positive low-titer samples (Cq > 30, Fig 3F). Even though the quality of this low-titer cohort was suboptimal due to re-using of swabs, we were able to decrease the false-negative rate for samples with cq > 30 from 96% (Fig 3B) to 60% (Fig 3F). It is important to note that the number of tested samples is very low and therefore it will be necessary to test larger cohorts to obtain robust numbers on false-negative rates. Moreover, additional parameters might require optimization such as determining the optimal sampling location (Nose swabs, throat swabs, sputum, or saliva) and the influence of swab type [22]. Though further improvements will be necessary to consistently detect SARS-CoV-2 RNA in unprocessed low-titer samples, bypassing the need for RNA purification dramatically accelerates and simplifies the evaluation of patient material.

As an additional asset of the simplified approach, we have observed that the endpoint of the high temperature RT-PCR with Volcano3G polymerase can also be easily evaluated on a blue-light LED screen, yielding strong green fluorescence for positive samples and allowing clear-cut differentiation by the naked eye (Fig 3G).

Together, the use of a heat-stable, RNA- and DNA-reading polymerase is poised to simplify detection of viral RNAs. By reducing handling steps, the chances of sample cross-contamination are minimized. Moreover, screening of unprocessed samples might be an economical way to identify highly contagious, asymptomatic individuals in population-wide screening campaigns, as high viral loads are present at the initial stage of infection [17]. The overwhelming dynamic spread of SARS-CoV-2 and the apparent bottle-necks in virus testing highlight the need for additional methodology that can be deployed in resource poor settings and that is operative in situations, where particular reagents such as RNA isolation kits might become scarce. Therefore, the direct detection of virus RNA from unprocessed patient samples and the ability to perform and read-out complex NAAT procedures using standard field PCR machines and blue-light screens could dramatically expand the COVID-19 testing capabilities and might also afford an important economic relief for health care providers in large parts of the world.

## Material and methods

### Patient samples and ethics statement

Nasopharyngeal swabs were collected at the COVID-19 test center hosted at the Klinikum Konstanz. Samples used in this study were procured with sterile dry swabs (Copan 160C Rayon, www.copaninnovation.com; Eurotubo collection swab 300253, www.deltalab.es; culture swab without transport medium 09-516-5009, www.nerbe-plus.de) and samples were processed within 24h for RNA isolation. Material eluted in RNAse/DNAse free deionized water not directly used for RNA isolation was stored at -20°C for further analysis. The study was approved by the local ethics committee of University of Konstanz (05/2020). The authors confirm that all research was performed in accordance with relevant guidelines/regulations and that informed consent was obtained from all participants and/or their legal guardians.

### RNA extraction

Nasopharyngeal swabs were eluted in 350 μl RNAse/DNAse free deionized water and 150 μl of the eluate was used for RNA isolation using the NucleoSpin 96 RNA kit (Macherey-Nagel, Düren, Germany), where the RA1 lysis buffer included in the kit was supplemented with 10 μg/ml of carrier RNA (Qiagen, Hilden, Germany) and 15 mM DTT. Automated handling of the samples was accomplished using the Integra Assist Plus pipetting robot (Integra, Biebertal, Germany). Finally, purified RNA was eluted into 60 μl RNAse/DNAse free deionized water.

### Generation of in vitro transcribed N1 amplicon

cDNA was generated from isolated viral RNA using the Maxima cDNA synthesis kit (Fermentas, Vilnius, Lithuania) according to the manufacturer’s protocol. A region corresponding to position 27923–28648 of the SARS-CoV2 genome (NC_045512.2) was amplified with Pfu DNA polymerase (primers Clone_N1_F and Clone_N1_R, see Table 1). The resulting 726 bp amplicon was purified, digested (KpnI/BamHI) and ligated in pBluescript-KS(+). The resulting plasmid was linearized with BamHI, purified and transcribed in vitro (T7 DNA polymerase, NEB) for 4 hours at 37°C to yield a 753 nt transcript. The purified transcript was treated with RNAse-free DNase (Roche) for 4 hours at 37°C and purified again (viral RNA isolation kit, Qiagen).

**Table 1:** Primer and probe sequences used in this study.

Molecular cloning		
Oligo name	Sequence	Final concentration
Clone_N1_F	5‘-TATAGGTACCCACAACTGTAGCTGCATTTCACC-3‘	500 nM
Clone_N1_R	5‘-TATAGGATCCGATATCAGCACCATAGGGAAGTCCAGC-3‘	500 nM
**Real time PCR**		
**Oligo name**	**Sequence**	**Final concentration**
CDC_N1-F	5’-GACCCCAAAATCAGCGAAAT-3’	670 nM
CDC_N1-R	5’-TCTGGTTACTGCCAGTTGAATCTG-3’	670 nM
CDC_N1-P	5’-FAM-ACCCCGCATTACGTTTGGTGGACC-BHQ1-3’	170 nM
RnaseP-F	5’-AGATTTGGACCTGCGAGCG-3’	670 nM
RnaseP-R	5’-GAGCGGCTGTCTCCACAAGT-3’	670 nM
RnaseP-P	5’-FAM-TTCTGACCTGAAGGCTCTGCGCG-BHQ-1-3’	170 nM
R2	5‘-GATCGCGCCCCACTGCGTTCTCCATTCTGG-3‘	250 nM

### Standard RT-PCR with purified RNA

Reference cq values were obtained using the Allplex 2019-nCoV Assay (Seegene, Seoul, South Korea) according to the manufacturer’s instructions. 8 μL of purified RNA were used to run 25 μl reactions on a Bio-Rad CFX96 and analysed with Seegene Viewer Software version 3.18.

### High temperature RT-PCR with purified RNA

Throughout this study high-temperature RT-PCR on RNA purified from either the in vitro transcribed viral genome fragment or the swab material was performed with the Volcano3G RT-PCR Probe 2x Master Mix (in short: Volcano3G) (myPOLS Biotec, Konstanz, Germany) using the CDC-approved N1 primer/probe mix from Integrated DNA Technologies (IDT, San Diego, CA, USA). In addition, sequence-identical primers and probe were obtained from Microsynth AG (Balgach, Switzerland) to produce data presented in S1 Fig. Typically, 10 μl reactions were set up containing 1 μl RNA, 1x Volcano3G MasterMix, CDC_N1 or RNaseP primer/probe mix (see Table 1) and 250 nM R2 primer. A three-stage thermal cycling program was performed on a Roche LightCycler 96, consisting of 1) 150 seconds at 75°C, 2) 10 cycles of 95°C for 5 seconds, 150 seconds at 75°C, 3) 45 cycles of 95°C for 10 seconds, 57°C for 35 seconds. All assays were performed in white low profile tubes with ultra-clear caps (ThermoFisher, Cat No: AB1771) as singleplex with FAM/BHQ1-labelled probes. Cq values were determined with Roche LightCycler Software 1.1.0.

### High temperature RT-PCR with unprocessed patient material

Nasopharyngeal swabs were eluted in 350 μl RNAse/DNAse free deionized water. These unprocessed samples were added directly to the PCR reaction. Reaction conditions were identical to those described for purified RNA as template, with the exception that the reaction volume was increased to 50 μl and 12 μl unprocessed sample was used (unless otherwise stated in the results section). In addition, successful amplification could be visualised by detection of the dequenched fluorescein signal (FAM) on a blue light transilluminator (Safe Imager 2.0, Invitrogen).

### Treatments for improving high-temperature RT-PCR of raw patient samples

In an effort to improve the efficiency of our protocol on unprocessed patient samples, several treatment regimens were tested. Nasopharyngeal swab samples in distilled water were supplemented with 1 ng/μl carrier RNA (Jena Analytik), 100 mM betaine (molecular grade, Sigma Aldrich), 0.05% bovine serum albumin (BSA) or a combination of all three, before adding the samples directly to the Volcano3G master mix. In another approach, swab samples were treated with 130 μg/μl nuclease-free proteinase K (Analytik Jena) for 10 minutes at 70°C in 5 mM HEPES (pH 7.4) followed by inactivation for 10 minutes at 95°C. Alternatively, samples were treated with 2.5 mM nuclease-free DTT (Thermo Fisher Scientific) for 10 minutes at 70°C or a combination of proteinase K and DTT. In the final optimized protocol (Fig 3F and S3 Fig) swabs were eluted in 50 μl nuclease-free water. A 10 μl aliquot was mixed with 1 μl (100 ng/μl) carrier RNA (Jena Analytik) and combined with 10 μl 2x proteinase K mix consisting of proteinase K (200 μg/ml, nuclease-free) and HEPES (pH 7.4, 20 mM) and incubated for 15 min at 45°C. Proteinase K was inactivated by addition of 2.2 μl phenylmethylsulfonyl fluoride (PMSF, 100 mM in DSMO). 13.8 μl of inactivated sample was used in a 60 μl RT-PCR reaction containing 30 μl Volcano3G (2x), 6 μl N1 primer/probe mix (IDT), R2 primer (250 nM) and MgCl_2_ (8 mM). The cycling parameters consisted of three stages: 5 min at 65°C, 10 cycles of 5 sec 95°C, 2.5 min 75°C, 45 cycles 15 sec 95°C, 45 sec 58°C.

### Limit of Detection (LOD) analysis

Serial dilutions of the in vitro transcribed amplicon were made in water containing 5 ng/μl RNA carrier mix (innuPREP Virus DNA/RNA Kit, Analytik Jena) and real-time PCR reactions (10 μl reaction volume) were set up as described above. Three to eight replicate reactions were conducted containing 1x10^6^, 1x 10^5^, 1x 10^4^, 1x10^3^, 100, 10, 5, 3 or 1 copies per reaction. To estimate the LOD, the fraction of positive reactions (cq < 40) was plotted against the log-transformed number of amplicon copies.

### Statistical analysis

Linear regression analysis was performed with Prism (GraphPad, La Jolla, CA, USA).

## Supporting information

S1 Fig The R2 reverse primer enhances detection of the viral N gene RNA. (PDF)

S2 Fig Swab-derived material contains inhibitory factors. (PDF)

S3 Fig The inhibitory effects of raw patient material can be ameliorated. (PDF)
