# Supplementary material for: Detection of SARS-CoV-2 from raw patient samples by coupled high temperature reverse transcription and amplification
Source: PLoS One. 2020 Nov 2;15(11):e0241740. doi: 10.1371/journal.pone.0241740 (PMC7605687; doi:10.1371/journal.pone.0241740)
Supplement: S1 Fig — (PDF) [file pone.0241740.s001.pdf]

Supplementary Figure S1

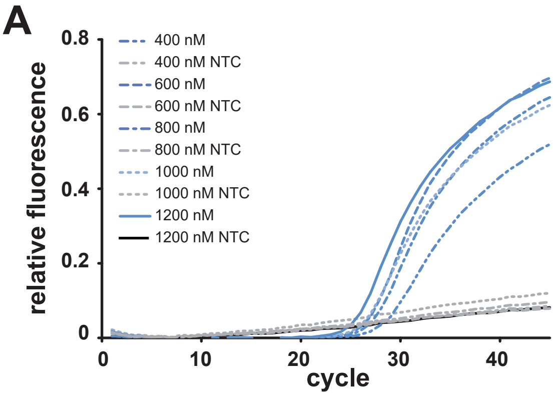

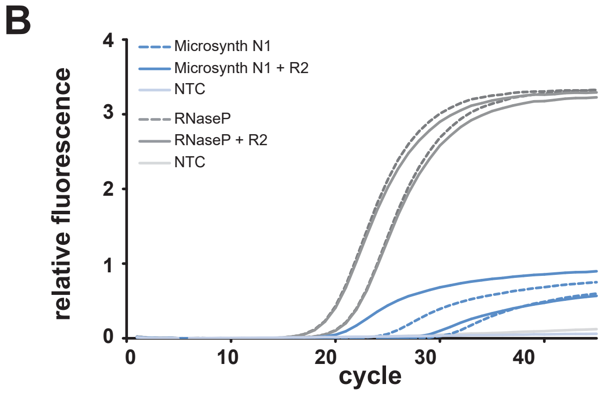

	final concentration per reaction [nM]				
**N1 forward primer**	400	600	800	1000	1200
**N1 reverse primer**	400	600	800	1000	1200
**N1 probe**	200	200	200	200	200
**Cq**	28.7	26.75	27.33	26.57	25.49

C

```
            1           10         20         30
SARS-CoV2   MSDNGPQ-NQRNAPRITFGGPSDSTGSNQNGERSGA
SARS_CoV1   MSDNGPQSNQRSAPRITFGGPTDSTDNNQNGGRNGA
OC43        MSFTPGKQSSSRASSGNRSGNGILKWADQSDQFRNV
HKU1        MSYTPGHYAGSRSSSGNRSGILKKTSWADQSERNYQ
MERS        MASPAAPRAVSFADNNDITNTNLSRGRGRNPKPRAA
229E        MATVKWADASEPQRGRQGRIPYSLYSPLLVDSEQPW
NL63        MASVNWADDRAARKKFPPPSFYMPLLVSSDKAPYRV
```

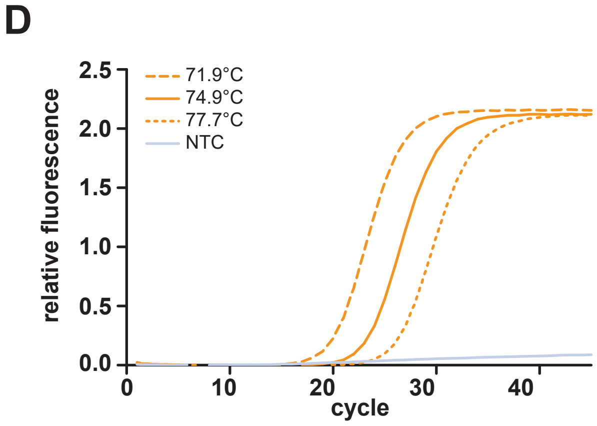

Supplementary Figure S1. The R2 reverse primer enhances detection of the viral N gene RNA.
A) N1 oligonucleotides and fluorescent probes according to CDC's recommendations were ordered from an alternative manufacturer (Microsynth AG) and assessed for their performance using Volcano3G and 500 copies of in vitro transcribed RNA encoding the N1 amplicon. Concentration of forward and reverse primers were adjusted for optimal performance. B) The chosen primer/probe concentrations were evaluated using isolated RNA from two confirmed SARS-CoV-2 positive patients, using RNAseP as control (dashed grey lines). Dashed blue lines: primer/probe pair without R2. Addition of R2 reverse primer at a final concentration of 250 nM enhanced the performance of the N1 primer pair, while showing no effect on RNAseP amplification (solid blue and grey lines, respectively). Light grey line: non-template control. C) Amino acid sequences of the amino-terminus of the beta-coronavirus N gene from SARS-CoV-2, SARS-CoV-1, MERS, and the human coronavirus strains OC43, HKU1, 229E, and NL63. Identical amino acids are marked in red. The N protein displays high sequence divergence at the amino terminus suggesting that the corresponding region of the N gene (upper row) is well suited for selective detection of SARS-CoV-2. D) Isolated RNA from a confirmed SARS-CoV-2 positive patient was analysed using the Volcano3G protocol and the N1 primer/probe mix from IDT in presence of 250 nM of R2. A temperature gradient was run during the reverse transcription reaction (step 1 and 2) of the Volcano3G thermocycling program.
