# Supplementary material for: Detection of SARS-CoV-2 from raw patient samples by coupled high temperature reverse transcription and amplification
Source: PLoS One. 2020 Nov 2;15(11):e0241740. doi: 10.1371/journal.pone.0241740 (PMC7605687; doi:10.1371/journal.pone.0241740)
Supplement: S2 Fig — (PDF) [file pone.0241740.s002.pdf]

Supplementary Figure S2

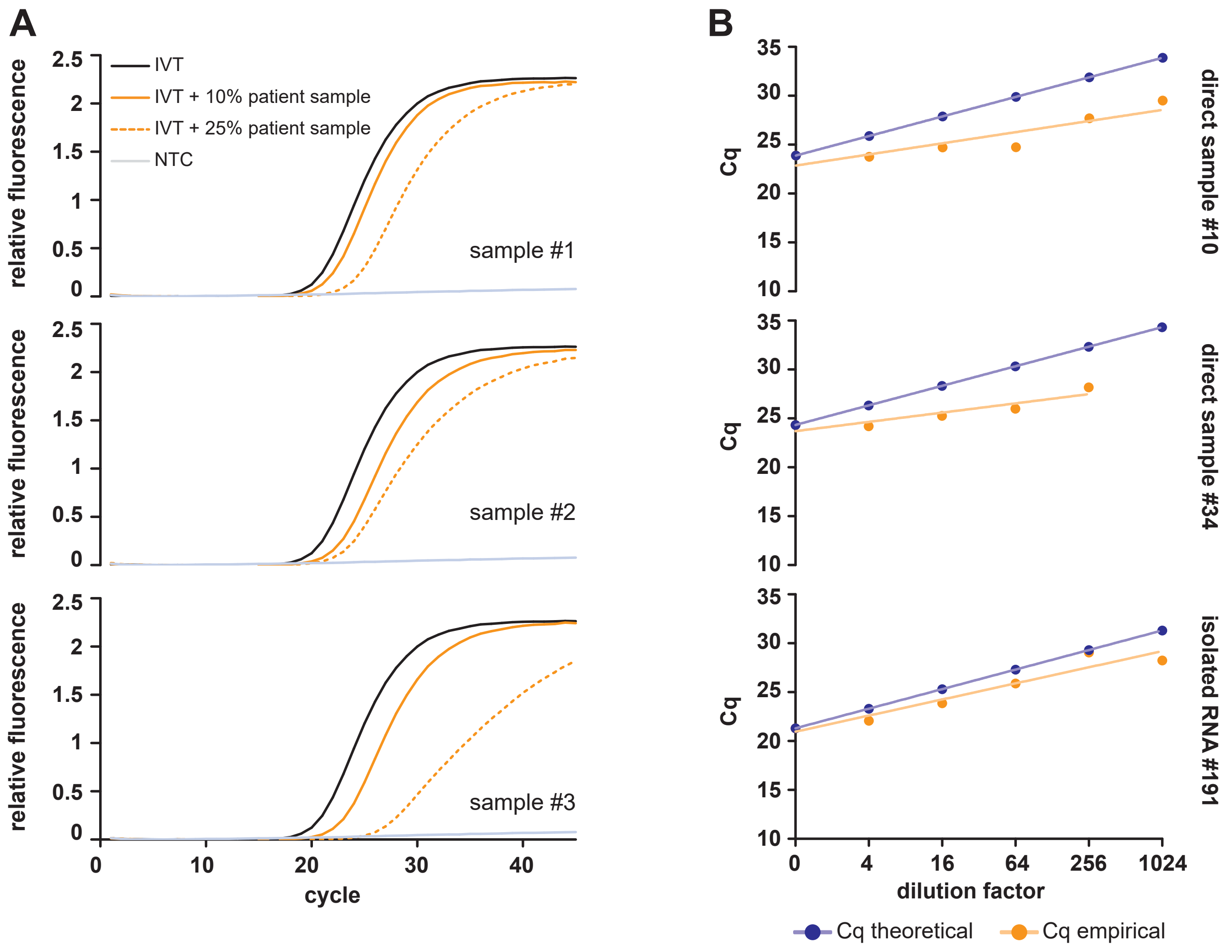

Supplementary Figure S2. Swab-derived material contains inhibitory factors.
A) Nasopharyngeal swab samples from three confirmed negative patients were serially diluted in RNAse free water and spiked with 5000 copies of in vitro transcribed RNA revealing the presence of PCR inhibitors. B) Unprocessed patient material from two confirmed SARS-CoV2 positive patients and isolated RNA from one confirmed positive patient were serially diluted in RNAse free water containing carrier RNA (1 ng/µl). Empirically determined Cq values are plotted against a theoretical dilution curve.
