# Supplementary material for: Detection of SARS-CoV-2 from raw patient samples by coupled high temperature reverse transcription and amplification
Source: PLoS One. 2020 Nov 2;15(11):e0241740. doi: 10.1371/journal.pone.0241740 (PMC7605687; doi:10.1371/journal.pone.0241740)
Supplement: S3 Fig — (PDF) [file pone.0241740.s003.pdf]

Supplementary Figure S3

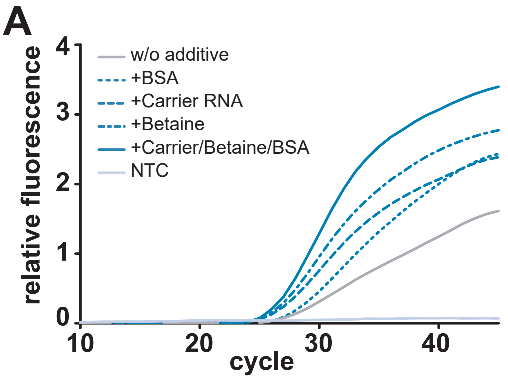

Additive	Cq	Cq gain
Carrier / Betaine / BSA	25,55	2,4
Betaine	25,78	2,1
Carrier RNA	26,01	1,9
BSA	27,51	0,4
RNAse free water	27,91	

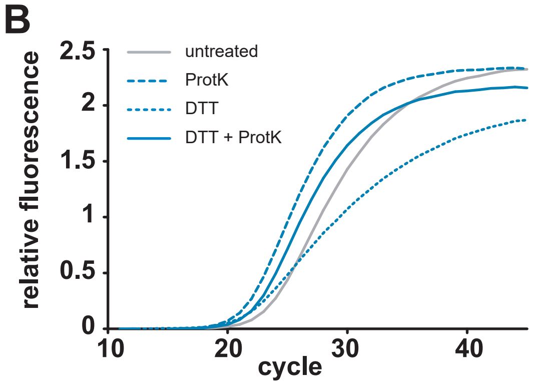

Additive	Cq	Cq gain
ProtK	19,6	1,7
DTT	19,9	1,4
DTT + ProtK	20,3	1,0
RNAse free water	21,3	

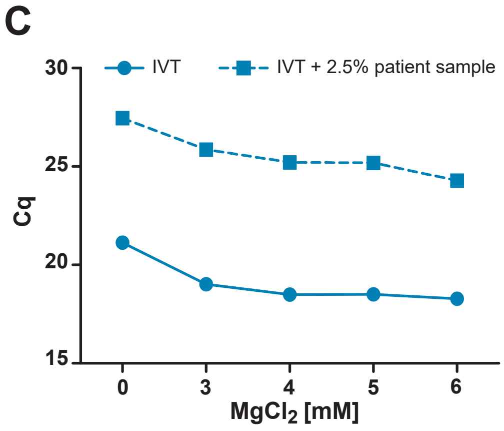

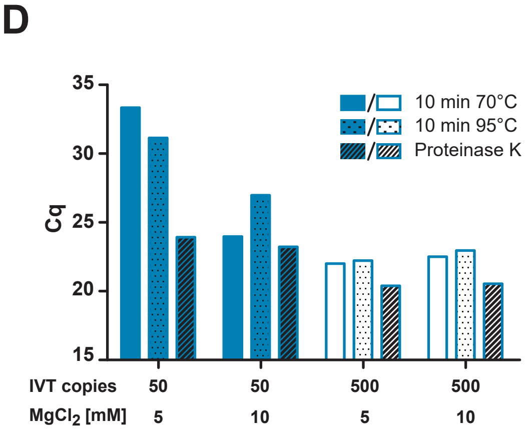

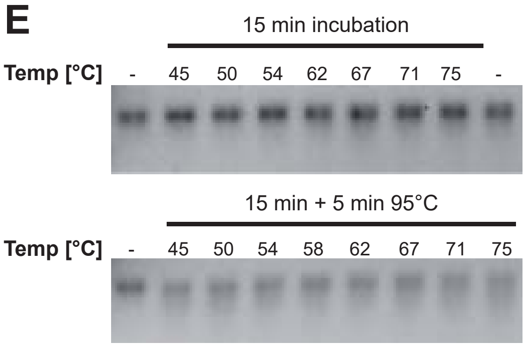

F

1) Swab in 50 µl RNAse-free H2O + 10 ng/µl carrier RNA

2) Mix 10 µl sample with 10 µl 2x proteinase K mix *(final: 100 µg/ml, 10 mM HEPES pH 7.4)*

3) Incubate 15 minutes at 45°C - Inactivate with PMSF *(final: 10 mM)*

4) Setup 60 µl final volume Volcano3G PCR:

PCR reaction:	
Volcano3G (2x)	30 µl
N1 primer/probe (IDT)	6 µl
R2 primer (25 µM)	0.6 µl
MgCl2 (50 mM)	9.6 µl
Sample	13.8 µl
**Total:**	**60** µl

Cycling parameters:		
65°C	5 min	
95°C	5 sec	10x
75°C	2.5 min	
95°C	15 sec	45x
58°C	45 sec	

Supplementary Figure S3. The inhibitory effects of raw patient material can be ameliorated.
A) An unprocessed nasopharyngeal swab sample of a confirmed SARS-CoV-2 positive patient was diluted in water plus either carrier RNA (1 ng/µl), betaine (100 mM), BSA (0.05%) or a combination of all three and subjected to Volcano RT-qPCR. B) Nasopharyngeal swab sample of a confirmed positive patient was diluted in water plus carrier RNA (1 ng/µl). Patient material was then treated with Proteinase K (ProtK, 130 µg/ml), DTT (2.5 mM) or a combination of both. Samples were incubated at 70°C for 10 min. Samples containing Proteinase K were additionally inactivated at 95°C for 10 min. C) Volcano3G reactions were performed with IVT template (1000 copies/rxn) in the presence or absence of 2.5% unprocessed nasopharyngeal swab sample (SARS-CoV-2 negative). Increasing amounts of MgCl2 (3, 4, 5, and 6 mM) were added to enhance detection. D) Nasopharyngeal swabs were dipped in 50 µl RNAse-free of which 40 µl was used for diagnostic PCR. The remaining 10 µl was mixed with 1 µl carrier RNA (100 ng/µl) and stored at -20°C. Five negatively tested samples were pooled and 10 µl aliquots were differentially processed. One aliquot was mixed with an equal volume of proteinase K mix (final concentration: proteinase K 100 µg/ml, HEPES pH 7.4 10 mM) and incubated 10 minutes at 70°C followed by 5 min at 95°C. Alternatively, aliquots were mixed with an equal volume of water and heated for 10 min at 70°C or 95°C. These processed samples were spiked into a Volcano3G reaction containing 50 or 500 IVT copies and 5 or 10 mM additional MgCl2. E) 75 ng IVT RNA in proteinase K mix (as in D) was incubated for 15 min at various temperatures (45°C-75°C) with (lower panel) or without (upper panel) an additional 5 min incubation at 95°C. F) Optimized protocol for detecting SARS-CoV-2 in nasopharyngeal swab samples without RNA isolation.
